# Radiotherapy treatment of adrenal gland metastases from hepatocellular carcinoma: clinical features and prognostic factors

**DOI:** 10.1186/1471-2407-14-878

**Published:** 2014-11-25

**Authors:** Le-Yuan Zhou, Zhao-Chong Zeng, Jia Fan, Bing Chen, Sheng-xiang Rao, Jian He, Ping Yang, Jia-zhou Hou, Zhi-feng Wu, Jian-ying Zhang, Yong Hu

**Affiliations:** Department of Radiation Oncology, The Affiliated Hospital of Jiangnan University (The 4th people’s hospital of Wuxi City), 200 Huihe Road, Wuxi, Jiangsu Province 214062 China; Department of Radiation Oncology, Zhongshan Hospital, Fudan University, 180 Feng Lin Road, Shanghai, 200032 China; Liver Cancer Institute, Zhongshan Hospital, Fudan University, 180 Feng Lin Road, Shanghai, 200032 China; Department of Radiology, Zhongshan Hospital, Fudan University, 180 Feng Lin Road, Shanghai, 200032 China

**Keywords:** Hepatocellular carcinoma, Adrenal gland, Metastasis, Radiotherapy, Prognosis

## Abstract

**Background:**

The optimal treatment for adrenal metastases from hepatocellular carcinoma (HCC) has not been established. This study analyzed the effects of radiation therapy (RT) for such metastases and identified clinical features and predictors of survival in these patients.

**Methods:**

We retrospectively investigated 55 patients with adrenal metastasis from HCC who had been treated with RT. Radiation doses to the adrenal lesions ranged from 26 to 60 Gy, while the intrahepatic lesions were treated by surgical resection, transarterial chemoembolization (TACE), liver transplantation, and/or RT. RT was conducted to adrenal lesions after their intrahepatic lesions were controlled more than 2 months. The parameters studied included survival rates and tumor responses to RT. The Kaplan-Meier method was used to evaluate survival rate and the Cox regression model was used to identify potential predictors of outcome.

**Results:**

The patients treated by RT had adrenal metastasis on the right side (41), the left (6), or on both sides (8). In all 55 patients, the median survival duration was 13.6 months and there was 100% pain relief after completion of RT. Adverse effects were mild to moderate. Unfavorable pretreatment predictors determined by univariate analysis were associated with multiple intrahepatic foci, metastases to additional organs, high γ-glutamyltransferase and alpha-fetoprotein levels, liver function of Child-Pugh classification B and uncontrolled primary HCC. By multivariate analysis, unfavorable predictors were multiple intrahepatic foci, metastases to additional organs and uncontrolled primary HCC.

**Conclusions:**

Radiotherapy as treatment for adrenal metastases in HCC is a good palliative therapy that is associated with reasonable safety. It appears reasonable that such patients should be considered to be treated with radiotherapy. Multiple intrahepatic foci, metastases to additional organs and uncontrolled primary HCC were unfavorable predictors.

## Background

Extrahepatic metastases occurring in hepatocellular carcinoma (HCC) are now observed more frequently because of the prolonged survival of HCC patients. Despite advances in treatment modalities and surgical techniques in recent years, the management of recurrence or distant metastasis remains a critical problem in treatment of HCC [1]. Intrahepatic recurrence can be controlled by several treatment modalities, such as repeated hepatectomy, transcatheter arterial chemoembolization (TACE), radiotherapy (RT), percutaneous ethanol injection therapy (PEIT), and liver transplantation [2, 3]. While treatment modalities of intrahepatic recurrence are well documented in the literature, there is much less information available that focuses on treatment strategies for extrahepatic metastasis. Although the adrenal glands are the second most frequent organ of extrahepatic metastasis from HCC, there are no definitive guidelines for treatment of these conditions [4–7]. In 2005, we retrospectively studied the effect of RT on the adrenal metastatic lesions in 22 HCC patients treated with two-dimensional conventional RT. With recent developments in radiotherapy technology involving precise delivery of focused high-doses on target volume, or sparing organs at risk, it is desired to update our understanding on the RT on the adrenal metastases in an expanded HCC cohort.

## Methods

### Source of patient and clinical data

A retrospective review of the medical records of 55 patients who underwent RT for adrenal metastases of HCC between January 2001 and August 2011 at the Zhongshan Hospital of Fudan University was performed. The patients’ characteristics from these records are summarized in Table 1. Approval for this study was obtained from the Zhongshan Hospital Research Ethics Committee.

**Table 1:** **Patients and tumor characteristics**

Independent variable	Patients	Kaplan-Meier survival	Univariate analysis	Multivariate analysis
	n	Median (months)	***P***	***P***
**Age (yr)**			.114	n.s.
≤50	23	12.63 ± 4.26		
>50	32	13.63 ± 4.88		
**Gender**			.411	n.s.
Male	52	13.63 ± 1.48		
Female	3	7.27 ± 0.63		
**γ-GT**	6 missing		.008	n.s
<150	33	17.80 ± 6.51		
≥150	16	5.57 ± 3.53		
**AFP level (μg/ml)**			.027	n.s
<400	33	15.90 ± 2.62		
≥400	22	5.57 ± 2.01		
**Child-Pugh classification**			.043	n.s
A	45	15.27 ± 1.63		
B	10	5.53 ± 2.43		
**Maximal diameter of intrahepatic tumors (mm)**			.074	n.s
≤80	33	15.13 ± 3.32		
>80	22	8.87 ± 1.46		
**Number of intrahepatic tumor(s)**			.000	.042
Solitary	45	15.90 ± 2.61		
Multiple (>2)	10	5.57 ± 3.30		
**Interval**			.422	n.s.
Synchronous	8	8.87 ± 1.22		
Metachronous	47	13.63 ± 1.52		
**Resection including liver transplantation for intrahepatic tumors**			.483	n.s.
Yes	35(4)	15.90 ± 2.38		
No	20	10.53 ± 3.17		
**Additional organ metastasis**			.001	.00
Yes	9	4.47 ± 0.45		
No	46	15.27 ± 1.63		
**Metastatic adrenal tumor size (mm)**			.476	n.s.
<50	21	15.13 ± 4.40		
≥50	34	13.20 ± 2.02		
**Location of adrenal lesion**			.614	n.s.
Single adrenal	47	13.63 ± 1.42		
Both	8	9.27 ± 5.33		
**Radiation dose (Gy)**			.102	.059
≥54	18	21.27 ± 8.46		
<54	37	12.93 ± 2.15		
**Primary HCC**			.00	.003
Controlled	38	17.80 ± 4.77		
Uncontrolled	17	9.77 ± 2.94		
**Response to RT**	5 missing		.017	n.s
PR	32	17.80 ± 8.28		
SD	18	12.63 ± 2.79		

### Diagnosis of HCC and adrenal metastasis

The diagnosis of HCC was confirmed by histology of a surgical specimen or by clinical diagnosis. For the latter, a positive diagnosis met the following criteria issued by the Chinese Liver Cancer Association in 1999 [8]. First, the serum alpha-fetoprotein (AFP) level should be >400 mg/l, to rule out patients with active liver disease, embryonal malignant teratoblastomas of the testes or ovary, or other malignant tumors metastasizing to the liver. Additionally, the tumor should have a characteristic appearance by one of several HCC imaging methods. Second, if the serum AFP level is <400 mg/l, the characteristic intrahepatic lesion should be confirmed by two imaging methods. To exclude patients with metastatic tumors from the digestive system or intrahepatic cholangiocarcinoma, the status of carcinoembryonic antigen or carbohydrate antigen 19.9 (CA19.9) should be negative for those with negative AFP levels. Of the patients in this study, 35 patients were confirmed by pathology and 20 patients were clinically diagnosed. All these patients had a Karnofsky performance scale score of at least 80. The Child-Pugh classification, reflecting liver function, was scored based on the levels of serum bilirubin and albumin, prothrombin time prolongation, presence or absence of ascites, and encephalopathy.

Adrenal gland metastasis was primarily detected by abdominal-pelvic enhanced computed tomography (CT) imaging (37 patients) and magnetic resonance imaging (MRI) (18 patients). A positive diagnosis was reached in cases in which the interval appearance of an adrenal mass on serial images was considered to represent metastatic disease [7]. Symptoms included back pain (32 patients) and epigastric/upper quadrant visceral pain (10 patients). Other symptoms such as hypertension, Cushing syndrome or electrolyte imbalance, which might relate to the abnormal function of the adrenal gland, were not observed in the cohort.

### Treatment of primary HCC

Intrahepatic primary tumors were treated with TACE alone (20 patients) or resection followed by TACE (31 patients). Surgical resection focused on the removal of only the intrahepatic primary tumors. The TACE procedure comprised a combination of targeting chemotherapy with various drugs, including 5-fluorouracil (5-FU), cisplatin (DDP), and mitomycin C (MMC). There were no standard protocols for the treatment. Most of these patients were treated two to six cycles of TACE. Four patients received liver transplantations. There were 3 patients in this cohort receiving target drug, sorafenib after adrenal metastasis irradiation.

### External beam radiotherapy (EBRT)

If the patients’ intra-hepatic lesions remained to be stable more than 2 months, radiotherapy to adrenal metastasis will be conducted. Intrahepatic lesions and adrenal metastasis were irradiated simultaneously in 3 patients. Patients received limited-field RT using a linear accelerator (SIEMENS) with a 6- or 15-MV (depending on tumor location and depth) photon beam strictly focused on the adrenal lesion. In our study, there were 29 patients treated before 2005 who received two-dimensional (2-D) conventional RT, and the prescribed dose was rarely more than 54 Gy. After 2005, there were 26 patients who were treated with three-dimensional conformal or intensity-modulated RT (3-D CRT or IMRT).

The patients were positioned in supine and immobilized using a vacuum bag to improve the reproducibility of daily treatments. If the adrenal metastatic lesions were adjacent to the liver, the patients received training in respiration to reduce the amplitude, increase the frequency and minimize tumor movement before the initiation of RT. The respiratory movement was estimated during simulation, and if it was >1.0 cm, abdominal compression was applied to minimize the movement [7]. Images for CT-based isocentric, multiportal, or 3-D CRT were taken with the patient lying in treatment couch. The CT scan slices were obtained at intervals of 3 mm from the superior to inferior extent of the simulated fields. CT images were directly transmitted to the 3-D planning system. The gross tumor volume (GTV) was defined as the volume of the radiographically visible adrenal lesion. The clinical target volume (CTV) was created by adding a 0.4-cm margin around the visible metastatic tumor. The planning target volume (PTV) was determined as the CTV plus 0.5-0.7 cm. The median tumor dose was 50 Gy (range of 26-62 Gy) in daily 2.0 Gy fractions, five times per week. Factors that indicated the need for a reduced dose were considered such as progressive primary disease, poor performance status, severe adverse effects, and patient inconvenience during EBRT. The scheduled doses depended on factors relating to field size and anatomic location. The organs such as spinal cord, liver, stomach, small bowl and kidney were the key consideration. Adverse effects, such as gastrointestinal reactions, the volume of irradiated kidneys, and the status of intrahepatic tumors and distant metastasis, were taken into account. The schedule prescribed doses were no less than 46 Gy. The normal tissue dose limitation are listed as follows: The maximum dose of the spinal cord did not exceed 45 Gy. The whole liver mean dose did not exceed 30Gy. The maximum dose of the small bowl did not exceed 54 Gy and V50 ≤ 5%, V40 ≤ 30%. The maximum dose of the stomach did not exceed 54 Gy and V45 < 50%. The mean dose of two kidneys did not exceed 15Gy and V20 ≤ 33%, V28 ≤ 20%, V12 ≤ 55%.

### Follow up and assessment of response and toxicity

Before treatment, an evaluation of medical history, physical examination, complete blood cell count, and liver and kidney function tests (Roche Diagnostics, Indianapolis, IN, USA) were performed. Clinical monitoring was performed every 1 - 2 weeks. After RT, the patients were periodically evaluated by CT or MRI in an outpatient clinic at our institute or at a hospital selected by the patient.

Patients were advised of the need for follow up 6 - 12 weeks after the completion of RT. Responses to RT were evaluated at that time by abdominal CT (enhanced or unenhanced) or MRI. Patients were monitored every 3 months thereafter. Local responses were classified according to the Response Evaluation Criteria in Solid Tumors with modifications [9].

Serum AFP levels were determined using an electrochemiluminescence immunoassay (Roche Diagnostics). For AFP-positive patients, this determination was repeated at the first follow-up visit. The changes in AFP levels were compared approximately 3 months apart, from pre-RT to 6 weeks after completion of RT. The threshold for an AFP decline was either a serum concentration of <20 mg/l or a >10% reduction in the serum level [10].

The visual analogue scale of pain was used to assist patients describing the intensity of pain experienced. On the numerical rating scale, the patient was asked to identify a position between 0 (no pain) to 10 (worst pain imaginable) [11]. Partial pain relief (partial response or PR) was defined as a decrease of the initial pain score by at least two points without increased analgesic dosage. Complete pain relief (complete response) was defined as a decrease to 0 on the pain scale without analgesic increase. Progression after response was defined as (1) an increase in pain with return to the initial pain score or higher without analgesic increase or (2) an increase in administration of an analgesic agent from a lower phase to phase 3 or 4, irrespective of the pain score. All of the patients in this study filled out a pain score form before and after treatment. Responses were evaluated within 1 week of completion of EBRT.

### Toxicity assessment

Acute and late reactions were scored according to the Common Terminology Criteria for Adverse Events version 3.0 (CTCAE) of the National Cancer Institute (USA) [12]. Complete blood cell counts and routine chemistry determinations were performed twice a week during the course of treatment.

### Statistical analyses

The overall survival period was defined from the date of diagnosis of adrenal metastases to the date of death or the last follow-up appointment. Kaplan-Meier curves were generated to analyze overall survival. The Cox regression model was used to detect associations between survival and serum AFP and γ-GT levels, intrahepatic tumor status (tumor size and number), Child-Pugh classification, and radiation dose additional organ metastasis, and primary HCC status (controlled or uncontrolled). Statistical analyses were performed using the Statistical Package for Social Sciences, version 13.0. *P* values <0.05 were considered statistically significant.

## Results

### Patient characteristics

The demographic characteristics of the study patients are listed in Table 1. The cohort included 52 men and 3 women with a mean age of 54.4 ± 11.0 years (range, 34 - 82 years). Pretreatment variables showing an effect on survival, including demographics, clinical laboratory tests, tumor characteristics, and treatment information for patients, are also listed in Table 1.

In total, 63 lesions in 55 patients received RT. However, for six of the lesions, CT or MRI documentation after RT was lacking, and therefore, the following data are expressed for the remaining 57 documented lesions. In 68.4% of the lesions (39/57), a PR (include 1 got CR) was achieved, and stable disease (SD) was reached in 31.6% (18/57). Serum AFP levels were abnormally high before RT in 40 patients. By comparison to pretreatment serum levels, an AFP decline was seen in 19 patients (47.5%), a stabilization was seen in 9 patients (22.5%), and 12 patients (30.0%) had increased AFP levels. Of all the patients, the median survival time was 13.6 months, and the 1-year survival rate was 58.7%, two-year survival rate was 32.3%. The 3 patients receiving target therapy, whose survival time were 12.6, 9.2, 24.1 months, respectively.

Before RT, there were 42 patients with pain that was always rated as mild on the visual scale (≤3) with or without treatment with non-narcotic analgesic agents. All patients experienced pain relief in different degrees after completion of RT (Table 2).

**Table 2:** **Dose effect for tumor volume and pain relief**

Radiation dose (Gy)	Patients (n)	Response to RT(PR) 5 missing	Pain relief
26	1	0/1	-
36	1	0/1	-
40-49	10	7/10	7/7
50	25	13/22	19/19
54-62	18	12/16	16/16

### Pretreatment variables and results of univariate and multivariate analyses

**Figure 1:**
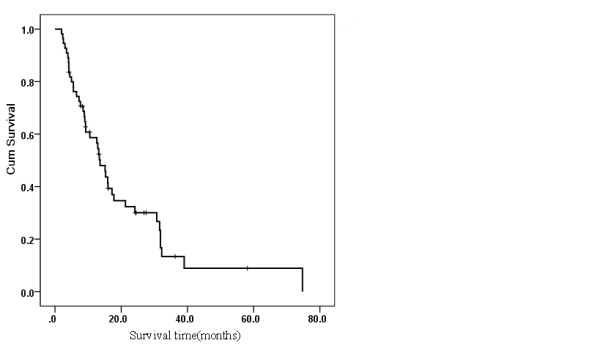
**Overall survival curves for the 55 patients in the study.**

The median survival time of the 55 patients was 13.6 months (Figure 1). Relative to pretreatment variables, the Kaplan-Meier survival analysis showed that the following variables were related to unfavorable overall survival: multiple intrahepatic tumors, higher serum AFP and γ-GT levels, liver function Child-Pugh classification B or higher, metastasis to additional organ(s), uncontrolled primary HCC, and a poor response to RT.

**Figure 2:**
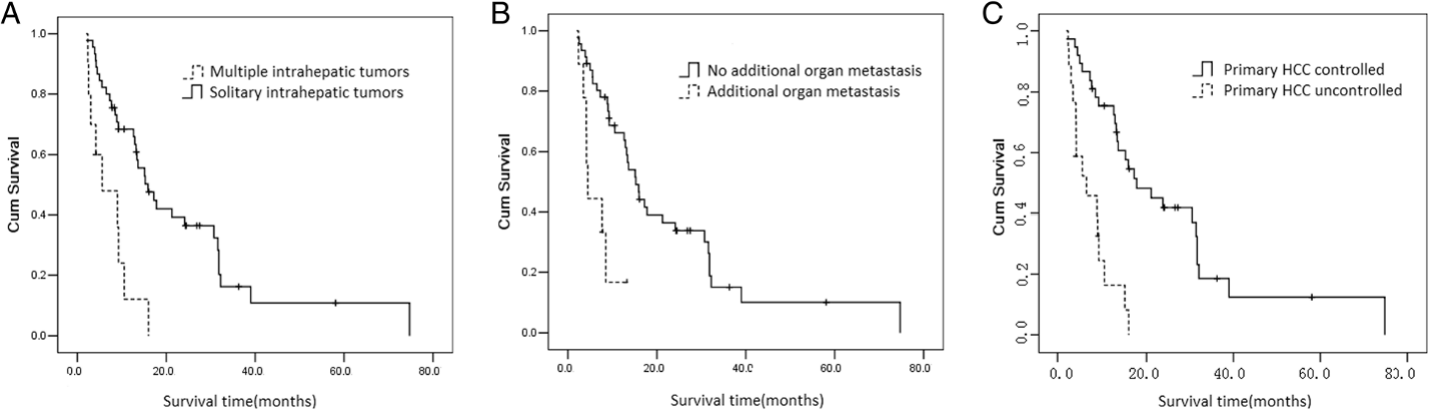
**Survival curves are shown based on solitary or multiple intrahepatic tumors (A), with or without additional organ metastasis (B), controlled or uncontrolled primary HCC (C).**

Multivariate analysis indicated that unfavorable pretreatment predictors were associated with multiple intrahepatic tumors (P = .042) (Figure 2A), metastasis to additional organ(s) (P = .00) (Figure 2B), and uncontrolled primary HCC (P = .003) (Figure 2C) as also shown in Table 1.

### Failure patterns

Four patients’ adrenal lesions (four lesions) relapsed after RT. The time of relapse ranged from 6 - 11 months (median time was 9 months) and the prescribed doses for treatment were 40 - 50 Gy. One patient received a second-course of RT and achieved PR.

At the time of the retrospective analysis, 42 of the patients died. However, none of these deaths resulted from adrenal metastasis-related complications. Liver failure caused by primary tumor progression (upper digestive tract bleeding and hydroperitoneum) resulted in 35 deaths. Other deaths were the consequence of lung metastasis (four patients), abdominal lymph node metastasis and related complications (two patients), and brain metastasis (one patient).

### Adverse effects

Side effects (acute/long-term toxicity) that were observed in patients during or after the RT treatment are listed in Table 3. The most acute common adverse effects were loss of appetite and nausea during the procedure. These effects usually occurred at the end of the therapy, and most patients did not need fluid infusion. Thrombocytopenia was observed in 32 patients and was associated with the presence of poorer liver function and hypersplenism. Incidence of acute side effects of 29 patients who received two-dimensional conventional RT is similar to those of patients received 3-D CRT or IMRT. One patient stopped to receive RT due to digestive tract bleeding after receiving 26 Gy radiation. One patient terminated RT because of low platelet count, and three patients terminated RT by themselves because of anorexia. Two patients had increased serum levels of liver enzymes that were usually less than three times the upper normal limit. The increasing of serum liver enzymes occurred during RT for one patient and 2 months after RT for the other patients. In the former, the prescribed dose was completed after a 2-week rest period. Mean liver doses were not excess 25 Gy for the two patients.

**Table 3:** **Side effects in 55 patients received radiotherapy (2D, 3D)**

Side effects RTOG	Grade			
	1	2	3	4
Acute toxicity				
Gastrointestinal				
Anorexia	25(15/29,10/26)*	5(3/29, 2/26)*	0	0
Diarrhea	6(3/29, 3/26)*	0	0	0
Vomiting	7(4/29, 3/26)*	0	0	0
Bone marrow suppression				
Leucopenia	19(11/29, 10/26)*	9(4/29, 5/26)*	0	0
Thrombocytopenia	11(6/29, 5/26)*	16(6/29, 10/26)*	5(2/29, 3/26)*	0
Late toxicity				
Liver injury	12(7/29, 5/26)*	5(6/29, 5/26)*	0	0
Gastrointestinal injury	15(9/29, 6/26)*	0	0	0
Kidney injury	3(2/29,1/26)*	0	0	0
Spinal cord injury	0	0	0	0

*Cases of the group with two-dimensional conventional radiotherapy before 2005 and those of three-dimensional conformal or intensity-modulated radiotherapy were displayed in the brackets, and no significant difference was found between the two groups.

Long-term toxicity including mild to moderate hepatic dysfunction(17/55), abdominal pain and diarrhea(15/55), transient albuminuria(2/55) and Mild elevation of blood urea nitrogen and creatinine(1/55) was observed in this cohort. Incidence of late side effects in patients who received 2-D conventional RT is also similar to those in patients received 3-D CRT or IMRT. No clinical symptoms of radiation-induced nephritis (hypertension, anemia, elevated blood urea nitrogen and creatine levels, or albuminuria), and digestive tract injury (intestinal perforation and stenosis)were observed more than 3 months after RT. Among the patients who survived longer than 2 years after RT, no long-term treatment-related morbidity was identified.

## Discussion

HCC has long been one of the most serious cancers in the world. From World Health Organization statistics in 2000, it has been estimated that there are at least 564,000 new cases of HCC per year globally. Especially in the Asia-Pacific region, HCC is a highly prevalent disease associated with high mortality [13]. Advances in diagnostic and multidisciplinary applications have enhanced the possibility of long-term survival for patients with the disease. Unfortunately, the frequency of distant metastasis has concomitantly increased, with major metastatic organs being the lung, lymph node, bone, and adrenal gland. Several factors affect metastasis, including tumor factors, tumor–stromal interaction, inflammatory and immune reactions, and so on. MMP-9 is crucially involved in tumor cell invasiveness metastasis [14, 15]. Radiation therapy for metastasis to lymph node, bone, and lung is safe and effective [11, 16, 17]. However, with regard to RT for adrenal metastasis, there are few published reports.

Surgical resection, TACE, and systemic chemotherapy are additional treatment options for adrenal metastasis. Table 4 lists the survival outcomes of various therapies for metastatic adrenal tumors from HCC. Because the adrenal gland has three feeding arteries (inferior phrenic artery, aorta, and renal artery), TACE treatment for adrenal metastasis is technically difficult and is associated with lower efficacy. HCC and its metastatic lesions are typically insensitive to chemotherapy. There have been several comparative studies of available treatment options. Park *et al.*[6] reported that 30 patients with well-controlled intrahepatic lesions and no additional organ metastasis other than adrenal glands, received treatments such as TACE, adrenalectomy, chemotherapy, and RT. The median survival times of patients after adrenalectomy, non-surgical treatment, and no treatment were 21.41, 11.05, and 5.64 months, respectively. Momoi *et al.* [5] analyzed 20 patients treated for adrenal metastasis of HCC by adrenalectomy, TACE, or PEIT and found that the median survival period was 10.2 months. No significant difference in cumulative survival rates was found among these three treatment groups.

**Table 4:** **Effect of various therapies for metastatic adrenal tumor from HCC**

Treatment	Author	Time	Case (n)	Tumor	Survival time (from Treatment of adrenal metastases)		
					Median (months)	Survival rate (1 year)	Survival rate (2 years)
Surgery	Momoi H [5]	2002	13	8/13 have additional organs metastases.		51.3%	42%
	Park JS [6]	2007	5	With well-controlled intrahepatic foci no additional organs metastases	21.4	100%	50%
				Single adrenal metastases			
TACE	Momoi H [5]	2002	4	With tumor thrombus or additional organs metastases	5.9, 6.5, 16.1, 21.3 months		
	Taniai N [18]	1999	2	Single adrenal metastases and well-controlled intrahepatic foci	Survived 3 and 8 months (still live)		
PEIT	Momoi H [5]	2002	4	additional organs metastases (1 case), intrahepatic tumor (3 case)	7.6, 8.5, 21.4, 32 months		
PEIT + TACE	Park JS [6]	2007	19	well-controlled intrahepatic foci, no additional organs metastases	10.5	43	0
RF+ TACE	Yamakado K [19]	2009	6	intrahepatic tumor (T3), additional organs metastases (3 cases), size of metastatic adrenal tumor (3-8 cm)	24.9		
No treatment	Park JS [6]	2007	6	well-controlled intrahepatic foci, no additional organs metastases	5.6	0%	-
RT	Zhou LY	2014	55	Patients of liver function Child-Pugh C were excluded	13.6	58.7%	32.3%

Here, we analyzed records of 55 patients treated with RT for adrenal metastasis from HCC. To the best of our knowledge, it is the largest study described to date. In this cohort, median survival time was 13.6 months (95% CI, 10.80–16.46 months) and average survival time was 20.7 months (95% CI, 14.5–27.0 months). There was no significant difference in median survival between the group that received a dose of more than 54 Gy, in which median survival was 21.3 months (95% CI, 4.7–38.0 months), and the group that received less than 54 Gy was 12.9 months (95% CI, 8.7–17.1 months) (*P* = 0.089). The tumor response rate was 64%, which reflects the sensitivity of adrenal metastasis from HCC to RT.

That whether RT for adrenal metastasis could alter the course of disease is not easily assessed, especially as randomized studies have not been performed. Based on present clinical data, we observed that RT could alleviate pain, and influence the level of serum AFP, thereby probably affect the progress of the disease. Definitive guidelines for treatment of adrenal gland metastatic tumor of HCC have not been established. It is difficult to answer the question as to whether RT really improved the survival of these patients. Greater overall survival rates in patients treated with RT were observed in this cohort compared with other reports, suggesting that RT may improved the prognosis of such patients. In a recent report from Korea [6], a median survival of 21.4 months was reported in HCC patients with adrenal metastases treated by adrenalectomy. However, only five patients receiving adrenalectomy were included in their study.

Prognostic factors for patients with adrenal metastases from HCC were analyzed based on tumor status, liver function, or treatment selection. The negative prognostic factors were identified by univariate analysis as tumor status (including multiple intrahepatic foci, additional organ metastases and primary HCC uncontrolled), higher serum γ-GT and AFP levels, and a Child-Pugh classification B. Because adrenal metastasis was rarely regarded as the direct death cause of HCC, RT was conducted to adrenal lesions after their intrahepatic lesions were controlled more than 2 months. A good response to RT was found to be a favorable prognostic factor and a relatively high dose of irradiation (≥54 Gy) tended to be a good factor in the present cohort. In selected patients based on the prognostic factor (controlled primary HCC), high-dose RT showed better clinical response (31.6 ± 6.5 *Vs* 15.3 ± 2.1 months, *P* < .05), though the numbers of subgroup patients were very small. In the other selected patients group based on adrenal-only metastases, and solitary intrahepatic foci, high-dose RT also seemed to demonstrate better clinical response, but no significant differences were observed probably due to the small number of patients.

Relapse of adrenal lesions after RT was found in four patients. The time of relapse ranged between 6 - 11 months and the prescribed doses were 40 - 50 Gy. Therefore, for some patients a relatively higher dose may be more appropriate. No clinical symptoms of radiation-induced nephritis were observed during or after RT and cases of increased serum levels of liver enzymes were not related to radiation dose. New techniques being developed, such as image-guided RT(IGRT) and stereotactic body RT (SBRT), enable a higher delivery dose to tumors relative to neighboring normal tissue. Casamassima *et al*. [20] and Chawla *et al*. [21] adopted SBRT for treatment of metastases to the adrenal glands in well-tolerated doses (the prescription dose was 36 Gy in three fractions and ranging from 16 Gy in four fractions to 50 Gy in 10 fractions). No consistent dose-response relationship was apparent for the palliative RT described.

Our results indicate the possibility that appropriate conditions of RT for adrenal metastasis from HCC may contribute to alleviate pain symptoms. In comparison with no treatment, there is a tendency to improved survival, especially in cases of well-controlled intrahepatic lesions and metastasis to no additional organs other than adrenal gland has occurred. Relatively higher doses of RT (greater than 54 Gy) are likely to prolong patient survival. Therefore, we propose RT as a treatment option aimed at local control in addition to adrenalectomy. However, a thorough evaluation of the efficacy of RT for adrenal metastases will require further studies in larger patient populations.

## Conclusions

Radiotherapy as treatment for adrenal metastases in HCC is a good palliative therapy that is associated with reasonable safety. Unfavorable pretreatment predictors identified were multiple intrahepatic tumors, metastasis to additional organ(s) and controlled primary HCC. In the RT-treated HCC patients with adrenal metastases, intrahepatic tumor progression was found to be the major cause of death. In selected patients with well-controlled primary HCC patients, high-dose RT demonstrated better clinical outcome. It appears reasonable that such patients should be considered receiving radiotherapy.
